# Intrahissian Anisotropy and Second Degree Type I Block 

**Published:** 2010-06-05

**Authors:** Jeffrey L Williams, David Lugg, Robert Gray, Douglas Hollis, Michelle Stoner

**Affiliations:** Good Samaritan Health System, Lebanon Cardiology Associates, 775 Norman Drive, Lebanon, PA 17042

**Keywords:** Intrahissian, Second Degree Block, Anisotropy, Pacemaker

## Abstract

A 79 year old female presents for evaluation of multiple episodes of witnessed syncope. Invasive electrophysiologic evaluation revealed evidence of both intrahissian Wenckebach and anisotropy. This is the first report documenting both phenomena in the same patient. The patient underwent a dual-chamber pacemaker implantation without complication.

## Case Description

A 79 year old female with past medical history of paroxysmal atrial fibrillation on sotalol, transient ischemic attack, hyperlipidemia, pulmonary embolus presented for further evaluation of her syncopal episodes. Over the past 4 years, the patient had experienced 11 episodes of syncope and often witnessed by her husband.  During these episodes, he found her to have blood pressure of 95/60 with a pulse of 80 and episodes occurred in both standing and sitting position.  She does not report chest pain, palpitations, or shortness of breath at these events. Echocardiogram and nuclear perfusion stress tests indicated no abnormalities other than diastolic dysfunction. Her surface electrocardiogram demonstrated sinus rhythm with PR interval of 130msec and QRS duration of 100msec.  Holter monitor was unrevealing.

**Figure 1:**
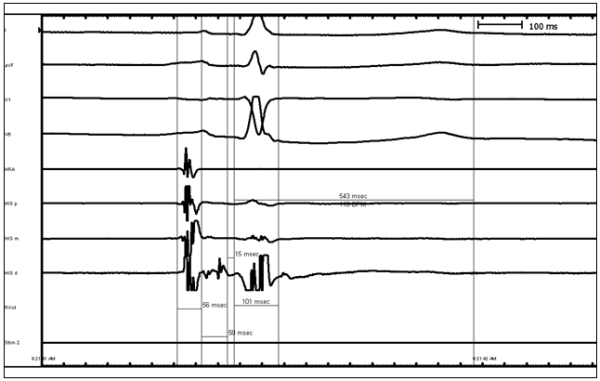
Baseline His Recording Demonstrating Split His Potential.

**Figure 2:**
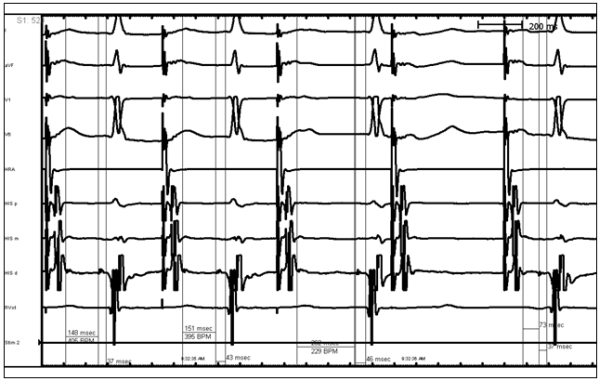
Atrial Extrastimuli Demonstrating Intrahissian Type I Second Degree Block.

**Figure 3:**
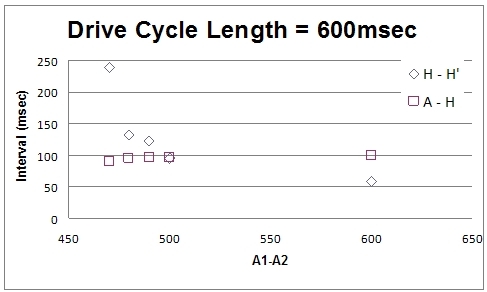
Discontinuity in the H-H' interval with atrial extrastimuli suggesting intrahissian anisotropy.

Electrophysiology study revealed sinus cycle length of 1070msec, PR interval of 163msec, QRS duration of 102msec, and QT interval mildly prolonged at 536msec. A split His potential was observed with duration of 58msec (Figure 1). Atrial pacing revealed apparent Wenckebach but closer inspection revealed intrahissian second degree type I block (Figure 2). At a drive cycle length of 520msec, progressive H-H' prolongation was observed with eventual H' drop.  The return cycle demonstrating a shortening of the H-H' interval. Atrial extrastimuli were delivered with a drive cycle length of 600msec and an H-H' jump of 105msec was obtained (Figure 3). Sinus node recovery times indicated no significant sinus node dysfunction. Given the multiple episodes of syncope and evidence of intrahissian conduction disease, the patient underwent a dual chamber pacemaker implantation without complication. Patient consented for this case report publication.

## Discussion

His bundle block is usually accompanied by AV nodal and bundle branch disease and the majority of patients are women with significant concomitant heart disease [1]. No surface recordings captured any bradyarrhythmias in this patient however, syncope is typical in patients with His block [1]. This case is remarkable because the patient had no systolic dysfunction, obstructive coronary disease, or mitral annular calcification. In addition, intrahissian blocks are most often manifest as 2:1 and third degree atrioventricular block [1,2]. In this case, the progressive H-H' prolongation prior to the eventual H' drop with the shortened H-H' interval of the return cycle is atypical. We hypothesize the discontinuity in the H-H' interval with atrial extrastimuli with no significant changes to the AH interval most likely indicates anisotropy (possibly longitudinal dissociation) within the His bundle. We refer to anisotropy as a direction-dependent variation in tissue resistivity of the His bundle. Conversely, the discontinuity in H-H' may be explained by variation in parasympathetic input to the His bundle [1]. Finally, there is no pathological and experimental correlation data available for scarring and anisotropy and longitudinal dissociation in his bundle alone without involvement of right bundle (in this case, there was no evidence of right bundle branch involvement). Intrahissian Wenckebach [3] and evidence of intrahissian longitudinal dissociation [4] (without involvement of the right bundle branch) are rare and this is first report documenting both phenomena in the same patient.
